# Pyrexia in a young infant – is height of fever associated with serious bacterial infection?

**DOI:** 10.1186/s12887-022-03264-8

**Published:** 2022-04-08

**Authors:** Tan Shi Rui Victoria, Ong Gene Yong-Kwang, Lee Khai Pin, Ganapathy Sashikumar, Chong Shu-Ling

**Affiliations:** 1 Department of Paediatrics, KK Women’s and Children’s Hospital Singapore, 100, Bukit Timah Road, Singapore, 229899 Singapore; 2 Department of Emergency Medicine, KK Women’s and Children’s Hospital, Singapore, Singapore; 3 Duke-NUS Medical School, Singapore, Singapore

**Keywords:** Fever, Infants, Serious bacterial infections

## Abstract

**Background:**

Febrile infants ≤ 90 days old make up a significant proportion of patients seeking care in the emergency department (ED). These infants are vulnerable to serious bacterial infections (SBIs) and early identification is required to initiate timely investigations and interventions. We aimed to study if height of an infant’s temperature on presentation to the ED is associated with SBI.

**Methods:**

We performed a retrospective chart review on febrile infants ≤ 90 days old presenting to our ED between 31^st^ March 2015 and 28^th^ February 2016. We compared triage temperature of febrile infants with and without SBIs. We presented sensitivity, specificity, positive and negative predictive values (PPV and NPV) of fever thresholds at triage. A multivariable regression was performed to study the association between height of temperature and the presence of SBI, and presented the adjusted odds ratio (aOR) with corresponding 95% confidence intervals (CI).

**Results:**

Among 1057 febrile infants analysed, 207 (19.6%) had a SBI. Mean temperature of infants with a SBI was significantly higher than those without (mean 38.5 °C, standard deviation, SD 0.6 vs. 38.3 °C, SD 0.5, *p* < 0.005). For temperature ≥ 39 °C, sensitivity, specificity, PPV and NPV for SBI was 15.5% (95%CI 10.8—21.1%), 90.4% (95%CI 88.2—92.3%), 28.1% (95%CI 21.1—36.3%) and 81.4% (95%CI 80.5—82.4%) respectively. The height of fever was consistently associated with SBI after adjusting for age, gender and SIS (aOR 1.76, 95% CI 1.32—2.33, *p* < 0.001). However, 32 (15.5%) infants with SBIs had an initial triage temperature ≤ 38 °C.

**Conclusions:**

A higher temperature at triage was associated with a higher risk of SBI among febrile infants ≤ 90 days old. However, height of temperature must be used in conjunction with other risk factors to identify SBIs in young infants.

## Introduction

Febrile infants younger than 90 days old form a significant proportion of patients seeking care in the emergency department (ED). A cross-sectional study estimated 2 million febrile infants ≤ 90 days old presenting to EDs in the USA over the recent decade (2007–2017) [1, 2]. Among young febrile infants, up to 10–15% of have a serious bacterial infection (SBI) defined as bacteraemia, meningitis or urinary tract infection (UTI) [3–5]. Young infants are at risk of SBIs due to an immature immune system, vaccination naivety against common infections and potential perinatal exposure to certain pathogens [6], and may develop complications including cognitive deficits, hearing loss and potential mortality [7, 8].

Currently, guidelines recommend febrile infants ≤ 28 days old to undergo evaluation for SBIs including a lumbar puncture for cerebrospinal fluid (CSF) cultures, urine cultures and blood cultures, and in specific cases, cultures from stool, fluid or tissue samples, followed by prompt administration of empiric broad spectrum antibiotic coverage [9, 10]. For older infants between 28–90 days old, those without clinical or analytical risk factors may not require such extensive evaluation to avoid invasive procedures and unnecessary anxiety [11, 12]. However as clinical symptoms differentiating minor febrile illnesses from SBI are often absent in young infants, many are still subjected to hospitalisation and invasive procedures [13].

Published literature has provided criteria to identify patients at low risk of SBIs, including the Rochester criteria (RC), Philadelphia criteria (PC), and Boston criteria (BC). Whilst fever (≥ 38 °C) is necessary for the application of these clinical assessment tools, height of fever is not identified to be an independent risk factor for SBI [14–17]. Hyperpyrexia defined as a rectal temperature of ≥ 41.1 °C, was previously reported to be associated with bacterial meningitis by McCarthy et al*.* in the 1970s. Most of these cases were attributed to *Haemophilus influenzae* type B (Hib), which substantially decreased following the introduction of the Hib vaccination in the 1980s [18]. Other studies published conflicting results regarding the relationship between the height of fever and presence of SBI in the paediatric population [19–21]. Aronson and Pantell et al*.* created clinical prediction tools which include height of temperature to identify well looking infants at risk of invasive bacterial infections (IBI)[22, 23]. Results from recent systematic reviews by Rosenfeld-Yehoshua et al*.* in 2018 and Davis et al*.* in 2019 have suggested that hyperpyrexia in young infants is suggestive of increased risk of SBIs [24, 25].

We aim to study the association between the height of fever at ED triage and a subsequent diagnosis of SBI.

## Methodology

### Study design and settings

This is a secondary analysis performed on data used to study heart rate guidelines in the prediction of serious infections [7]. A retrospective analysis of febrile patients under the age of 90 days presenting to KK Women’s and Children’s Hospital Emergency Department was carried out between 31^st^ March 2015 and 28^th^ February 2016.

Our ED is the larger of two specialty paediatric emergency departments in Singapore accommodating approximately 150,000 children who attend annually [7].

### Eligibility

Febrile infants ≤ 90 days old were included in this study. All infants have their axillary temperatures screened at triage. If the temperature was ≥ 37.5^0^C, this infant would be included in the study. If the infant was wrapped in multiple layers of clothes, excess clothing would be removed. We included infants whose temperatures remained ≥ 37.5 °C and were eventually hospitalized. Excluded infants were those whose caregivers declined admission as there would be no follow up data available. Patients who were admitted but did not have any further evaluation for sepsis inpatient were also excluded. An example of such a situation would be an infant who examined well throughout admission and did not have any further episodes of fever in the hospital.

### Variables—triage

Axillary temperatures were measured using a Terumo digital thermometer at triage. Heart rate and blood pressure readings were automated measurements using the Dinamap GE ProCare 200 Vital Signs Monitor. Respiratory rate was manually measured by the triage nurse [7]. A locally validated severity index score (SIS) based on respiratory effort, colour, activity, temperature and play was also documented for all patients [26]. The maximum SIS score is 10 (stable), followed by 7–9 (moderately sick), and < 7 which potentially indicates a clinically ill child. Patients in our ED were consequently triaged into different acuity: Priority 1 (severely ill), 2 + (moderately ill) and 2 (stable) where they had to be reviewed by a doctor immediately, within 15 min or within the hour respectively.

### Variables – investigations

The following investigations were performed following admission in the well-looking infant who had 2 separate readings of fever ≥ 38.0 °C (including the temperature from triage) or a single temperature reading of ≥ 38.5 °C, or earlier if there were clinical findings of an unwell infant: full blood count (FBC) – looking specifically at the total white blood cell (WBC) count, absolute neutrophil count (ANC), haemoglobin levels, platelets, and the C-reactive protein (CRP). Bacteraemia was defined as bacterial growth in blood cultures (excluding common contaminants such as coagulase-negative staphylococcus). Urine for analysis and culture were routinely performed. Urinary tract infections were defined as a positive urine culture with a growth of a single bacteria of more than 10^5^ colony forming units (CFU)/L from a mid-stream urine sample, or 10^4^ CFU/L from a catheterized specimen [5, 27]. Lumbar puncture, CSF analysis and culture were performed when clinically indicated as decided by the attending physician. The diagnosis of bacterial meningitis was made following a positive CSF culture [5], or if the CSF fluid was suggestive of a bacterial meningitis such as significant pleocytosis (> 1000 cells/mm^3^), low CSF glucose or low CSF glucose to serum ratio (< 0.4), high CSF protein levels (> 100 mg/dL), in the absence of a positive culture. We included the latter because bacteria growth from CSF culture depends on the amount of bacteria present (which may be transient) and other supportive biochemical features on CSF analysis are vital for the diagnosis of bacterial meningitis [28, 29]. In specific cases, cultures would be obtained from stool, wounds or abscesses. Nasopharyngeal aspirate (NPA) samples were also obtained from infants with respiratory symptoms to test for common viral respiratory infections. In infants with hemodynamic instability or evidence of shock, resuscitation with fluid resuscitation, consideration for inotropic support and emergency intravenous antibiotics were initiated in the ED [30].

### Outcomes

SBIs were defined as bacteraemia, bacterial meningitis and urinary tract infections (UTI). IBIs were defined as bacteraemia and/or bacterial meningitis [23]. These diagnoses were based on ICD-10 diagnostic codes and cases reviewed manually by trained investigators in the initial data collection of the primary study [7]. Severity of illness was extrapolated from data such as the requirement for fluid resuscitation, inotropic support, mechanical ventilation and intensive care support.

All febrile infants ≤ 90 days old were hospitalized in our institution and monitored in the ward for at least 48 h. These infants were discharged following resolution of fever for 24 h and only after clinical assessment to be stable.

### Statistical analysis

Data were keyed into Microsoft Excel and analysed using IBM SPSS Statistics v26.0. Categorical variables were presented in frequencies and percentages while continuous variables were presented as mean (and standard deviation, SD), or median (and interquartile range, IQR), depending on normality. To study the association between temperature as a continuous variable and the presence of SBI, we used the student t-test (for normal data) and the Wilcoxon rank sum (if the data were non-normal).

To study any association between individual risk factors and the presence of SBI, we presented point estimates and their 95% confidence intervals (CI) for both the univariate regression (using odds ratio, OR) and multivariable regression (using adjusted odds ratio, aOR). In the multivariable regression, we chose variables that were significant on univariate analysis as well as variables routinely available at triage including age, gender, temperature and SIS. Temperature was analysed as both a continuous variable and a dichotomous variable with an a priori threshold at ≥ 39℃. Sensitivity, specificity, positive and negative predictive values (PPV and NPV) were also evaluated for different temperatures. Statistical significance was taken as *p* < 0.05.

### Ethics

Ethics approval for the primary study was granted by the SingHealth Institutional Review Board (IRB), with waiver of informed consent (2017/2680).

## Results

**Fig. 1:**
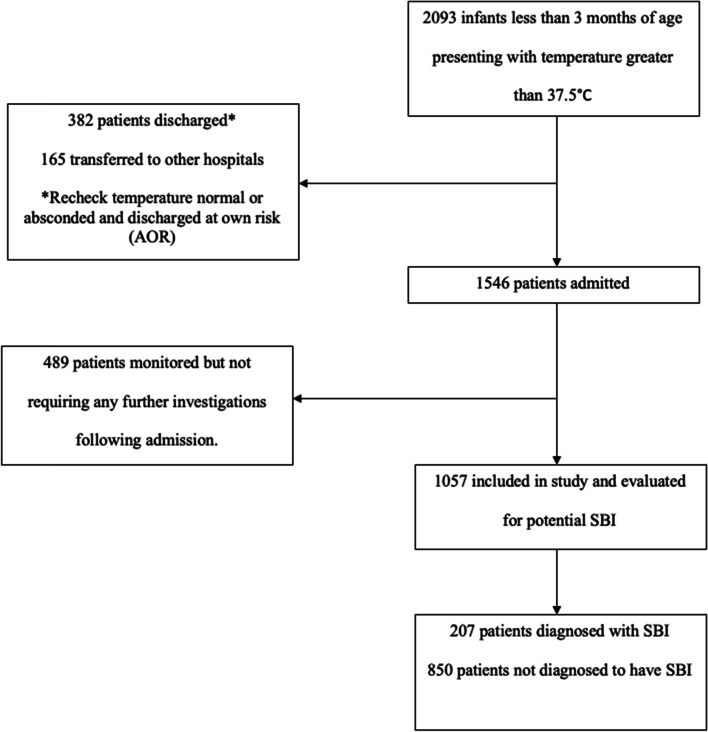
Flow diagram of study participants

Among 2093 infants screened, 1546 (73.9%) were hospitalized and 1057 (50.5%) were included in the analysis (Fig. 1). In this cohort, 82 (7.8%) were triaged as Priority 1 and 826 (78.1%) as Priority 2 + in the ED. Demographics and initial presentation of our study population are presented in Table 1. There were 207 (19.6%) with SBIs and 33 (3.1%) with IBIs. There was no significant difference in the median age of infants with SBI and those without (median 1.2 months, IQR 1.2–2.4, *p* = 0.434). There were a total of 247 (23.4%) infants ≤ 28 days, and among them 42 (17.0%) diagnosed with a SBI. Of the included patients, 42 (4.0%) infants had significant cardiac lesions, genitourinary anomalies and other congenital or genetic abnormalities, of which 9 (21.4%) were diagnosed to have a SBI on admission.

**Table 1:** Demographics and presentation of febrile infants in the emergency department

Demographics	SBI (*N* = 207)	NO SBI (*N* = 850)	*P* value
Median age in months (IQR)	1.2 (1.2–2.4)	1.2 (1.2–2.4)	0.434
Age ≤ 28 days (%)	41 (19.8)	207 (24.4)	0.177
Mean temperature in ℃ (SD)	38. 5 (0.6)	38.2 (0.5)	< 0.001
Male gender (%)	164 (79.2)	465(54.7)	< 0.001
Pulse rate per minute (SD)	168 (22.0)	166 (20.0)	0.237
Respiratory rate per minute (SD)	42 (6.7)	42 (6.4)	0.711
SIS < 7 (%)^A^	11 (5.3)	30 (3.5)	0.331
**Body Temperature Distribution (%)**			
< 38.0℃	32 (15.5)	212 (24.9)	0.040
38.0—38.9℃	143 (69.1)	556 (65.4)	0.317
39.0—39.9℃	30 (14.5)	76 (8.9)	0.017
> 40.0℃	2 (1.0)	6 (0.7)	0.698
**Triage Category (%) **^**B**^			0.081
1 (%)	23 (11.1)	60 (7.1)	
2 + (%)	161 (77.8)	665 (78.2)	
2 (%)	23 (11.1)	125 (14.7)	
**Interventions received (%)**			
Fluid bolus (%)	22 (10.6)	78 (9.2)	0.522
Inotropic support (%)	3 (1.4)	3 (0.4)	0.060
Ventilatory support (%)	4 (1.9)	13 (1.5)	0.582
ICU admission (%)	6 (2.9)	8 (0.9)	0.027

^A^The severity index score – used to identify a sick child based on the respiratory effort, colour, activity, temperature and ability to play. Scores < or equal to 7 indicate a very sick child

^B^The hospital’s triage system reflecting the urgency at which patients need to be reviewed. 1- seen immediately; 2 +—seen within 15 min of triage, 2- seen within first hour of triage

The mean temperature was higher in an infant with SBI compared to one without (38.5℃, SD 0.6 vs. 38.3℃, SD 0.5, *p* < 0.001). Among infants diagnosed with a SBI, 32 (15.5%) had first presented to the triage with a temperature less than 38 °C. For temperature ≥ 38 °C associated with SBI, we found a sensitivity of 84.5% (95%CI 78.9—89.2%), specificity 24.9% (95%CI 22.1—28.0%), PPV 21.5% (95%CI 20.4—22.7%) and NPV 86.9% (95%CI 82.5—90.3%). There were 114 (10.8%) patients who presented to the paediatric emergency department with a temperature ≥ 39 °C and of these infants, 32 (28.1%) had a SBI. Accounting for pyrexia ≥ 39 °C, sensitivity was 15.5% (95%CI 10.8—21.1%), specificity 90.4% (95%CI 88.2—92.3%), PPV 28.1% (95%CI 21.1—36.3%) and NPV 81.4% (95%CI 80.5—82.4%). A single infant (0.1%) presented to triage with hyperpyrexia. This infant did not have a SBI and was discharged after 2 days of hospitalization.

Of the infants with a SBI (Table 2), 181 (87.4%) were diagnosed with a UTI. *Escherichia coli* (*E.coli*) (*n* = 105, 57.1%) and *Klebsiella pneumonia* (*n* = 13, 7.1%) were the most likely bacterial pathogens. This was followed by 21 infants (9.2%) infants who were diagnosed with bacteraemia, of which *E.coli* (*n* = 9, 42.8%) and *S. agalactiae* (*n* = 6, 28.6%) were the most common. There were 16 patients diagnosed with bacterial meningitis (*n* = 21, 9.2%) amongst which 3 patients had *E.coli* and 2 had *S. agalactiae* meningitis. The other cases were treated as bacterial meningitis based on abnormal CSF fluid analysis. Of those diagnosed with a SBI, 12 (5.8%) had more than one positive culture, most frequent being a UTI with concurrent bacteraemia (*n* = 6, 50.0%), followed by bacterial meningitis with bacteraemia (*n* = 4, 33.3%) In patients with a UTI, 149 (82.3%) were older than 28 days of age.

**Table 2:** Characteristics of patients, temperature and management stratified by the different SBI

Type of SBI	UTI (*n* = 181)	Bacterial meningitis (*n* = 16)	Bacteraemia (*n* = 21)
Male gender (%)	146 (80.7)	12(75.0)	16 (76.2)
Mean temperature℃ (SD)	38.5 (0.6)	38.6 (0.6)	38.4 (0.655)
Need for fluid resuscitation (%)	14 (7.7)	3 (18.6)	8 (38.1)
Need for inotropic support (%)	1 (0.6)	0	2 (9.5)
Need for ICU care (%)	3 (1.7)	1(6.3)	2 (9.5)
Most common pathogens	*E.coli* *K. pneumoniae*	*E.coli* *Streptococcus agalactiae*	*E.coli* *Streptococcus agalactiae*

In those with a SBI (Table 1), fluid resuscitation was required in 22 (10.6%), inotropic support required in 3 (1.4%) and 6 (2.9%) were admitted to the intensive care unit (ICU). UTI was the commonest SBI (*n* = 3, 50%) amongst those admitted in the ICU. The median length of stay in ICU was 2.0 days (IQR 1.0–9.3) for infants with a SBI compared to 3.0 days (IQR 1.0–8.5) for infants without a SBI (*p* = 0.842). One patient death was documented secondary to bacteraemia complicated by fulminant liver failure. This patient had underlying trisomy 21 with pulmonary hypertension.

As shown in Table 3, the univariate analysis showed that temperature (*p* = 0.017) and gender (*p* < 0.001) were statistically significant for SBI. In the multivariable analysis, a temperature of ≥ 39 °C was associated with an increased risk of SBI (aOR 1.62, 95% CI 1.02 – 2.56, *p* = 0.040), after accounting for gender, age and SIS. When analysed as a continuous variable, we found that the height of the temperature was consistently associated for SBI after adjusting for age, gender and SIS (aOR 1.76, 95% CI 1.33—2.33, *p* < 0.001).

**Table 3:** Univariate and categorical multivariate analysis of temperature trend and risk for SBI

	Univariate analysis Unadjusted OR (95% CI)	*P* value	Multivariable analysis Adjusted OR (95% CI)	*P* value
Temperature^C^	1.71 (1.10–2.67)	0.017	1.62 (1.02 – 2.56)	0.040
Male gender	3.16 (2.20–4.54)	< 0.001	3.11 (2.16 – 4.47)	< 0.001
Age < 28 days	0.88 (0.53 to 1.13)	0.178	0.88 (0.59 – 1.29)	0.498
SIS < 7	1.44 (0.69–3.00)	0.334	1.17 (0.54 – 2.52)	0.687

^C^Defining temperature ≥ 39℃

Of note, 370/1057 (35.0%) of infants in our study population did not undergo a lumbar puncture. However, infants who did not undergo a lumbar puncture and in whom no source of fever found were monitored until at least 24 h afebrile prior to discharge to ensure that a SBI would not be missed.

## Discussion

In this single-centre study, we described the relationship between the height of fever at ED triage and risk of SBI among infants ≤ 3 months of age. High temperature as a continuous variable, as well as a temperature threshold of ≥ 39 °C, were independently associated with increased risk of SBI after accounting for gender, age and SIS.

The presence of pyrexia is important in the early recognition of serious infections in young infants. In diagnostic algorithms, the height of temperature is not consistently specified [12–15]. For the past few decades, there have been conflicting reports on the association between high temperature and SBIs [17–19]. In older age groups, height of fever alone is unreliable in predicting SBI [21]. A prospective cohort study in paediatric patients aged 0–5 by Williams et al*.* suggested that whilst higher temperatures were associated with a SBI, a third risked being overlooked if they did not reach a temperature threshold of 38 °C [27]. There were also other studies suggesting that pyrexia ≥ 39–40 °C may be associated with SBI particularly in young infants [22, 30–32]. After adjusting for age, gender and SIS, we similarly demonstrated that a temperature ≥ 39 °C was an independent predictor for SBI. However, we recognize that temperature is a dynamic vital sign, and height of fever should be interpreted in conjunction with other risk factors.

The prevalence of SBI varies widely across international literature. Our study cohort (*n* = 228, 21.5%) reported a relatively high prevalence of SBI [33]. From a health services perspective, this may be because we are the larger of only 2 paediatric EDs locally. Young infants with borderline temperatures and who examine well at primary health services would likely be discharged and not referred to our hospital. Whilst SBIs are reported to be more prevalent in infants ≤ 28 days [5–7, 30], our study population demonstrated a greater proportion of SBI in the older infants > 28 days old (*n* = 166, 80.2%). These findings may be different because of a higher proportion of booked pregnancies with effective antibiotic prophylaxis for mothers with known *S. agalactiae* colonization locally [34] and subsequently there were only 6 (2.6%) positive cultures for GBS infections documented during this study period. Nonetheless, this emphasizes the importance of careful clinical evaluation in a febrile infant aged between 28–90 days. Higher temperatures ≥ 39 °C were demonstrated high specificity and NPV for SBI.

*E.coli* was the most frequently isolated bacterial pathogen in our study population, and *E.coli* UTI was the most commonly identified SBI. These results were epidemiologically similar to other studies [2, 35]. In infants with a concurrent UTI and bacteraemia, most were > 28 days of age. This suggests that there still is a need for thorough evaluation for bacteraemia in infants > 28 days of age, in the presence of a diagnosed UTI.

We recognise the limitations of this study. Whilst rectal thermometry is gold standard for temperature measurement in infants, we used axillary temperature because it is easy to measure and avoids the risk of rectal injury [36]. In this retrospective study, investigators were trained to perform structured data entry using a specific template to minimize inaccuracies and bias in data collection. We acknowledge limitations in using EMR diagnostic codes to derive the study population because infants with SBIs may have been misdiagnosed. A more comprehensive approach including examination of all microbiology results could avoid potential inaccuracies and should be utilised for future research. Although not every patient underwent complete evaluation for lumbar puncture, urinalysis and culture and blood tests, all patients were monitored until afebrile for at least 24 h prior to discharge to ensure that a SBI was not missed. For the diagnosis of bacterial meningitis, we included patients whose CSF fluid analysis was suggestive of a bacterial meningitis in the absence of positive CSF culture [28, 29]. We acknowledge that this may result in over-diagnosis of bacterial meningitis but in doing so we accounted for the possibility of culture negative bacterial meningitis. There was a small proportion of infants with a temperature above 40℃, which limited our data for hyperpyrexia in this population. Our chosen temperature threshold of ≥ 39℃ was decided a priori and we recognise that future research should utilize data-driven thresholds. We only studied the outcome of SBI, however we know that serious viral infections like enterovirus and herpes simplex virus may cause encephalitis, meningitis and viremia [37]. From the potential subjects, 489 were not evaluated for further SBI on admission and were excluded from our analysis. Finally, this study was performed at a single institution in Singapore in Asia, which may limit its relevance in other international centres.

## Conclusions

Our study has revealed some implications when assessing febrile infants in the ED. We found that a higher temperature may confer a greater risk for SBI. However, height of fever should be interpreted in conjunction with other risk factors to identify infants at risk for SBIs. Repeated monitoring and clinical assessment is necessary.

## Data Availability

The de-identified datasets used and analysed during this study are available from the corresponding author on reasonable request.

## Abbreviations

ANC: Absolute neutrophil count
CFU: Colony forming unit
CRP: C-reactive protein
CSF: Cerebrospinal fluid
ED: Emergency department
EMR: Electronic medical records
NPA: Nasopharyngeal aspirate
IBI: Invasive bacterial infection
SBI: Serious bacterial infection
SIS: Severity index score
UTI: Urinary tract infection
WBC: White blood cells

## References

[1] Ramgopal S, Aronson PL, Marin JR (2020). United States' Emergency Department Visits for Fever by Young Children 2007–2017. West J Emerg Med..

[2] Powell, Elizabeth C., et al. "Epidemiology of bacteremia in febrile infants aged 60 days and younger." Annals of emergency medicine 71.2 (2018): 211–216. 10.1016/j.annemergmed.2017.07.488 PMC5815881 28988964

[3] Baraff LJ, Oslund SA, Schriger DL, Stephen ML (1992). Probability of bacterial infections in febrile infants less than three months of age: a meta- analysis. Pediatr Infect Dis J.

[4] Baker MD (1999). Evaluation and management of infants with fever. Pediatr Clin North Am.

[5] Kuppermann N, Dayan PS, Levine DA (2019). A Clinical Prediction Rule to Identify Febrile Infants 60 Days and Younger at Low Risk for Serious Bacterial Infections. JAMA Pediatr.

[6] Huebner RE, Mbelle N, Forrest B, Madore DV, Klugman KP (2002). Immunogenicity after one, two or three doses and impact on the antibody response to coadministered antigens of a nonavalent pneumococcal conjugate vaccine in infants of Soweto. South Africa Pediatr Infect Dis J.

[7] Chong SL (2018). "A retrospective review of vital signs and clinical outcomes of febrile infants younger than 3 months old presenting to the emergency department.". PloS one.

[8] Hui C, Neto G, Tsertsvadze A, et al. Diagnosis and Management of Febrile Infants (0–3 Months). Rockville (MD): Agency for Healthcare Research and Quality (US); 2012 Mar. (Evidence Report/Technology Assessments, No. 205.)Available from: https://www.ncbi.nlm.nih.gov/books/NBK92690/

[9] National Collaborating Centre for Women's and Children's Health (UK). Feverish Illness in Children: Assessment and Initial Management in Children Younger Than 5 Years. London: Royal College of Obstetricians & Gynaecologists (UK); 2013 May. PMID: 25340238. 25340238

[10] Baraff LJ, Bass JW, Fleisher GR, Klein JO, McCracken GH, Powell KR, Schriger DL (1993). Practice guideline for the management of infants and children 0 to 36 months of age with fever without source. Agency for Health Care Policy and Research. Ann Emerg Med..

[11] Hamilton JL, John SP (2013). Evaluation of fever in infants and young children. Am Fam Physician.

[12] Pantell RH, Roberts KB, Adams WG, Dreyer BP, Kuppermann N, O'Leary ST, Okechukwu K, Woods CR (2021). Subcommittee on febrile infants. Evaluation and Management of Well-Appearing Febrile Infants 8 to 60 Days Old. Pediatrics..

[13] Dorney K, Bachur RG (2017). Febrile infant update. Curr Opin Pediatr.

[14] Jaskiewicz JA, McCarthy CA, Richardson AC (1994). Febrile infant collaborative study group. febrile infants at low risk for serious bacterial infection–an appraisal of the Rochester criteria and implications for management. Pediatrics..

[15] Baker MD, Bell LM, Avner JR (1993). Outpatient management without antibiotics of fever in selected infants. N Engl J Med.

[16] Baskin MN, O’Rourke EJ, Fleisher GR (1992). Outpatient treatment of febrile infants 28 to 89 days of age with intramuscular administration of ceftriaxone. J Pediatr.

[17] Gomez, Borja, et al. "Validation of the “step-by-step” approach in the management of young febrile infants." Pediatrics 138.2 (2016). 10.1542/peds.2015-4381 27382134

[18] McCarthy PL, Dolan T (1976). Hyperpyrexia in children. Eight-year emergency room experience. Am J Dis Child..

[19] Surpure JS (1987). Hyperpyrexia in children: clinical implications. Pediatr Emerg Care.

[20] Alpert G, Hibbert E, Fleisher GR (1990). Case-control study of hyperpyrexia in children. Pediatr Infect Dis J.

[21] Press S (1994). Association of hyperpyrexia with serious disease in children. Clin Pediatr.

[22] Pantell RH, Newman TB, Bernzweig J (2004). Management and Outcomes of Care of Fever in Early Infancy. JAMA.

[23] Aronson PL, Shabanova V, Shapiro ED, Wang ME, Nigrovic LE, Pruitt CM, DePorre AG, Leazer RC, Desai S, Sartori LF, Marble RD, Rooholamini SN, McCulloh RJ, Woll C, Balamuth F, Alpern ER, Shah SS, Williams DJ, Browning WL, Shah N, Neuman MI (2019). Febrile Young Infant Research Collaborative A Prediction Model to Identify Febrile Infants ≤60 Days at Low Risk of Invasive Bacterial Infection. Pediatrics..

[24] Rosenfeld-Yehoshua N, Barkan S, Abu-Kishk I, Booch M, Suhami R, Kozer E (2018). Hyperpyrexia and high fever as a predictor for serious bacterial infection (SBI) in children-a systematic review. Eur J Pediatr..

[25] Davis J, Lehman E (2019). Fever Characteristics and Risk of Serious Bacterial Infection in Febrile Infants. J Emerg Med.

[26] Ganapathy S, Yeo JG, Thia XHM, Hei GMA, Tham LP (2018). The Singapore Paediatric Triage Scale Validation Study. Singapore Med J..

[27] Soh S, Jamuar S, Puthucheary J. The baby bear book : a practical guide on paediatrics. Singapore : Red Cells Series, 2015

[28] van de Beek D (2016). ESCMID guideline: diagnosis and treatment of acute bacterial meningitis. Clin Microbiol Infect.

[29] Tacon CL, Flower O (2012). Diagnosis and management of bacterial meningitis in the paediatric population: a review. Emerg Med Int.

[30] Wynn James L, Wong Hector R (2010). Pathophysiology and treatment of septic shock in neonates. Clin Perinatol..

[31] De S, Williams GJ, Teixeira-Pinto A (2015). Lack of accuracy of body temperature for detecting serious bacterial infection in febrile episodes. Pediatr Infect Dis J.

[32] Carmon L, Goldbart A, Greenberg D, Ben-Shimol S (2017). Serious Bacterial Infections in Hospitalized Febrile Infants in the First and Second Months of Life. Pediatr Infect Dis J.

[33] Herr SM, Wald ER, Pitetti RD (2001). Enhanced urinalysis improves identification of febrile infants ages 60 days and younger at low risk for serious bacterial illness. Pediatrics.

[34] Lee J, Naiduvaje K, Chew KL, Charan N, Chan YH, Lin RT, Yong EL (2021). Preventing early-onset group B streptococcal sepsis: clinical risk factor-based screening or culture-based screening?. Singapore Med J..

[35] Greenhow T, Hung YY, Herz AM (2014). The changing epidemiology of serious bacterial infections in young infants. Pediatr Infect Dis J.

[36] Temperature measurement in paediatrics (2000). Paediatr Child Health.

[37] Hayakawa I (2020). Incidence and aetiology of serious viral infections in young febrile infants. J Paediatr Child Health..

