# Correction: Pyrexia in a young infant – is height of fever associated with serious bacterial infection?

**DOI:** 10.1186/s12887-022-03374-3

**Published:** 2022-06-15

**Authors:** Victoria Shi Rui Tan, Gene Yong-Kwang Ong, Khai Pin Lee, Sashikumar Ganapathy, Shu-Ling Chong

**Affiliations:** 1 Department of Paediatrics, KK Women’s and Children’s Hospital Singapore, 100, Bukit Timah Road, Singapore, 229899 Singapore; 2 Department of Emergency Medicine, KK Women’s and Children’s Hospital, Singapore, Singapore; 3 Duke-NUS Medical School, Singapore, Singapore

**Correction: BMC Pediatr 22, 188 (2022)**

**https://doi.org/10.1186/s12887-022-03264-8**

Following publication of the original article [1], the authors reported an error in the author list: the article had published with the authors’ respective given, middle and family names incorrectly ordered. The names have since been corrected in the published article and the correctly ordered names may be seen in the author list of this erratum.
